# The HIV epidemic and prevention response in Tigrai, Ethiopia: a synthesis at sub-national level

**DOI:** 10.1186/1471-2458-14-628

**Published:** 2014-06-20

**Authors:** GebreAb Barnabas, Elisabetta Pegurri, Hiwot Haile Selassie, Warren Naamara, Samuel Zemariam

**Affiliations:** 1 Initiative for Health Development in Africa, PO Box 1016069, Addis Ababa, Ethiopia; 2 UNAIDS, Country office, Economic Commission for Africa, Old Building, PO Box 5580, Addis Ababa, Ethiopia; 3 UNAIDS, Economic Commission for Africa, Old Building, PO Box 5580, Addis Ababa, Ethiopia; 4 Tigrai Health Bureau, HIV/AIDS Office, Mekelle, Ethiopia

**Keywords:** HIV, Acquired immunodeficiency syndrome, Evidence based policy, Vulnerable populations, Priority setting

## Abstract

**Background:**

This study, the first of its kind carried out at sub-national level in Ethiopia, was conducted in order to understand the dynamics of HIV transmission at regional and district level in Tigrai, Ethiopia; and to assess the adequacy of the HIV prevention response.

**Methods:**

Routine data from health centres, data from available published and grey literature and studies, and primary qualitative information were triangulated to draw an updated picture of the HIV epidemic, HIV response and resource allocation in Tigrai.

**Results:**

HIV prevalence in Tigrai was 1.8% in 2011 (EDHS). ANC data show that there has been a continuous decline in the prevalence of HIV in both urban and rural areas (urban: 14.9% in 2001 to 5.0% in 2009; rural: 5.2% in 2001 to 1.3% in 2009, ANC surveillance data). Variability in prevalence by zone and by district was observed. Possible reasons for higher prevalence include the presence of mobile seasonal workers, highly urbanized centres, a high concentration of economic activity and connecting roads and large commercial farms. Sex workers, seasonal farm workers and HIV negative partners in discordant couples were identified as being at higher risk. There is no evidence that programme planning is done on the basis of geographical variations in HIV prevalence and there are gaps in programmes and services for certain high risk population groups.

**Conclusion:**

Considerable efforts have been invested in the HIV prevention response in Tigrai however, these efforts do not fully respond to the actual needs. For a more effective and targeted HIV prevention response, studies and data syntheses need to be carried out at sub-national level in order to accurately identify local specificities and plan accordingly. Resources should be targeted towards areas where transmission is linked to sex work, mobility and the mobile labour workforce.

## Background

The “know your epidemic, know your response” (KYE-KYR) – or HIV Synthesis - is a systematic review of the epidemiology of HIV and the HIV response. The rationale is to better understand the heterogeneity of the HIV epidemic and to determine the degree of alignment between HIV prevention resource allocation and where and how transmission is occurring [1]. In some countries, the HIV Synthesis has been complemented by the Modes of Transmission model [2-6] which estimates the distribution of new HIV infections by key population groups.

**Figure 1:**
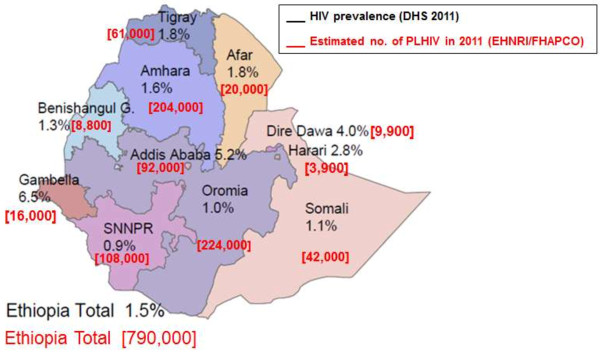
HIV prevalence and number of PLHIV by region in Ethiopia (FHAPCO, EHNRI, 2012).

According to the latest Ethiopian Demographic and Health Survey (EDHS 2011), HIV adult prevalence (15–49 years) was estimated at 1.5% (1.9% among women versus 1.0% among men; 4.2% urban versus 0.6% rural) [7]. However, a synthesis of HIV epidemiological data carried out in 2008 reported that the HIV epidemic in Ethiopia was heterogeneous with marked regional differences (Figure 1) and concluded that HIV programmes should not be led by national level statistics but instead targeted at districts or communities with higher prevalence; thereby requiring that research and data use is conducted at district level [8]. The same was confirmed by the 2014 Ethiopia National HIV Synthesis [9]. The Indian sub-continent also offered a recent example of the benefit of utilizing data from different sources at a more granular or local level for planning and tailoring the HIV response accordingly [10].

Thus this study, the first KYE/KYR synthesis performed at regional level in Ethiopia, was conceived by the Tigrai Health Bureau’s HIV Prevention and Control Office (HAPCO) and set out to see whether or not inferences reported at the national level could be confirmed for Tigrai region; one of the eleven regions of Ethiopia located in the northern part of the country. Furthermore, it aimed to assess the status of the epidemic in order to understand the adequacy of the HIV response.

The specific objectives of the KYE/KYR exercise in Tigrai were i) to describe and analyse the pattern and dynamics of the epidemic (KYE), ii) describe and analyse the prevention programme, policy environment and resource allocation (KYR), iii) to synthesize and link the epidemic and response data, and iv) recommend improvements in policies, programmes and the allocation of resources. When such objectives are realized, it is believed that policies become more relevant, resources used more fairly and efficiently, and programmes more effective than otherwise.

## Methods

### Search strategy for literature review

The search strategy of the literature review was: i) to look for any published material on HIV and AIDS in Ethiopia and Tigrai using the broadest terms of HIV and/or AIDS and Tigrai in PubMed, Medline and Cochrane Reviews. We further reviewed the Bibliography on HIV/AIDS in Ethiopia [11] and references lists of identified studies ii) unpublished documents were then collated from the Federal HIV Prevention and Control Office (FHAPCO) and the Tigrai Health Bureau, from NGOs working on HIV/AIDS in Tigrai and Masters level dissertations prepared by Addis Ababa University students on HIV/AIDS in Tigrai; iii) special effort was made to look for any information on high risk population groups such as sex workers, youth related behavioural studies, seasonal workers, alluvial gold miners, uniformed services, refugees, prisoners, long distance truck drivers, university students, discordant couples, street children, orphans and vulnerable children, men having sex with men and injecting drug users.

### Quantitative data analysis

#### Know your epidemic synthesis

Secondary data analysis of antenatal surveillance data, demographic health survey data for 2005 and 2011 and health facility data (including Prevention of mother to child transmission (PMTCT) or routine antenatal care (ANC) HIV testing data, voluntary HIV counselling and testing (HCT) and provider initiated counselling and testing (PICT)) was performed (see Table 1 for details). These data were analysed to assess trends in HIV prevalence over time, by geographical location, sex and age group. In addition routine data from health facilities were analysed/tabulated according to potential risks factors for HIV in the areas where health facilities are located such as urban (major towns)/rural location, proximity to transport corridors, presence of refugees, gold miners and seasonal workers in the catchment area. Since the Ethiopian fiscal year is split across two calendar years from June to July the reporting period for all health facility data corresponds to this (e.g. from June 2011 until July 2012). Data from the EDHS was analysed to investigate trends in HIV prevalence, knowledge, attitudes and sexual behaviour in Tigrai region compared with the rest of Ethiopia. In addition HIV related projections based on modelling carried out using EPP/SPECTRUM (version 4.47) software which utilises DHS, ANC surveillance and programmatic data [12] were obtained and used to estimate the number of people living with HIV in Tigrai as well as ART and PMTCT needs.

**Table 1:** Key secondary data sources for the synthesis of HIV epidemiological data for Tigrai

**Data source**	**Description**
***Demographic and Health Surveys (DHS)***	DHS (with an HIV component with seroprevalence, knowledge and behavioural data) at two time points for residents of Tigrai in 2005 and 2011 were available. DHS data were sourced from MEASURE DHS. All prevalence and percentages provided are weighted (weights provided by DHS/Macro). All p-values are from tests comparing weighted proportions.
**Antenatal Sentinel Surveillance**	From unlinked anonymous surveys based on HIV testing performed on left over blood collected for routine syphilis testing at antenatal clinics for the period 2001 to 2009 was available from the Ethiopian Health and Nutrition Research Institute. Data specimens were collected from 11 sites in Tigrai in 2009 (seven rural and four urban).
***Routine Prevention of Mother-to-Child Transmission (PMTCT) Testing Data***	Compiled and availed by the Tigrai Health Bureau. 101 health facilities in Tigrai were mapped and classified as urban or rural and according to risk factors present in their catchment areas. Data was available at health facility level for the period 2008/09 to 2010/11.
***Routine HIV Counselling and Testing (HCT) and Provider Initiated Counselling and Testing (PICT) Data***	Compiled by the Tigrai Health Bureau for the period 2008/09 to 2010/11 were analysed. HCT and PICT data were available at district level.

Data from the above sources were extracted, referenced and where possible triangulated. Data was managed in Microsoft Excel 2003 and analysed using SPSS software. Differences in proportions were analysed using χ^2^ tests and odds ratios and ninety-five per cent confidence intervals were calculated.

#### Qualitative research

Thirty focus group discussions (FGDs) were held with male and female members of identified high risk population groups (see Table 2 for details) using semi structured questionnaire guides. Discussion themes included knowledge, attitude and practice about HIV/AIDS, individual and structural risk factors that render various groups at risk of HIV infection in the region and access to HIV related services and gaps to be addressed. This data was utilised to assess factors directly associated with HIV transmission and was triangulated with routine PMTCT and HCT data at the zonal and district level to identify likely pockets of risk.

**Table 2:** Composition of the focus group discussions conducted as part of the qualitative component of the study

**Group**	**Location**	**Numbers**	**Comments**
**Youth**	Mekelle, Mai Chew & Adua	3 FGDs – 29 females	Age range 13–28 years; mean age 20.6 years for males and 19.4 years for females.
		3 FGDs – 30 males	
**Female sex workers**	Mekelle, Alamata, Mekhoni, Mai Chew & Setit Humera	6 FGDs	Included only women who self-identified as sex workers. Substantial proportion of sex workers < 20 yrs of age.
**Alluvial miners**	Mai Hanse of Asgede Tsimbla district	1 FGD – 12 participants	Convenience sample.
**Refugees**	Mai Ayni & Shimelba camps	2 FGDs – 12 males and 12 females	Interviews also with health officials and the camp social worker to complement FGD findings.
**Prisoners**	Mekelle & Tafie prison centres (Alamata)	2 FGDs – 8 individuals from each prison	Interviews also with prison officials and head of the prison clinic.
**People living with HIV**	Mekelle, Setit Humera & Alamata	6 FGDs – male and female – 8–12 participants in each FGD	Participants were people living with HIV (PLHIV) association members selected in consultation with team members from HAPCO.
**Seasonal migrant workers**	Ahferom and Weree Leke districts, Tsegede district	2 FGDs – 9 males in each group	Age range of participants 18–39 years with an average of 25 years. Convenience sample.
		2 FGDs – 12 males and 12 females	Included people who relocated spontaneously or as part of a government sponsored settlement programme (settlers).
**Armed forces**	Three border military camps	3 FGDs – 28 males	Included military personnel deployed in Tigrai.

FGDs = Focus group discussions HAPCO = HIV Prevention and Control Office, Tigrai.

In addition 33 key informant interviews (KIIs) using a semi-structured guide were also held with i) health service providers, ii) HIV and AIDS prevention and control board members, iii) youth, people living with HIV (PLHIV) and women’s groups and iv) local non-governmental organisations (NGOs) and international NGOs involved in the prevention and control of HIV in Tigrai. The questions focused on: activities and measures taken by government, partners and the communities involved in the HIV response; availability and access to services and supplies related to HIV prevention and treatment; and gathering evidence to substantiate responses. The FGD discussions and the KI interviews were transcribed and then translated to English. The topics and questions in the guides were used as themes/sub-themes and ideas/opinions related to each theme/sub-theme were coded under respective themes and subthemes. Ethical approval for the study was granted by the Tigrai Health Bureau, Ethiopia. The free and informed consent of all participants interviewed for the study was obtained.

#### Know your response synthesis

The response analysis consisted of a systematic review of published and grey literature including programme reports, HIV policies, technical guidelines, strategic plans and monitoring and evaluation reports both at national and regional level. Additional information was gathered through KIIs with HIV managers and service providers as explained above.

## Results and discussion

### HIV Prevalence and trends in Tigrai

Projections based on antenatal surveillance, EDHS 2005 and 2011 and programmatic data estimate there are approximately 61,000 PLHIV in Tigrai in 2011, of which 14,800 are children (0–14 years old) [13]. Adult HIV prevalence in 2011 was estimated at 1.8% [14].

EDHS 2011 data demonstrates a slight decrease in overall adult HIV prevalence in Tigrai over the past six years but this was not statistically significant (1.8% in 2011 vs. 2.1% in 2005, odds ratio (OR) 0.9, *p* = 0.73) and in both the male and female population (females: 2.2% in 2011 vs. 2.6% in 2005, OR 0.9, *p* = 0.78; males 1.3% in 2011 vs. 1.6% in 2005, OR 0.9, *p* = 0.88). There were no significant differences between HIV prevalence in Tigrai and the rest of Ethiopia (Tigrai: 1.8% 95% confidence interval (C.I.) 1.4-2.4 in 2011; Ethiopia excluding Tigrai: 1.4%, 95% C.I. 1.2-1.7 in 2011). According to the most recent ANC sentinel surveillance data (with several data points to enable trend analysis unlike DHS) there has been a continuous decline in the prevalence of HIV in both urban and rural areas in Tigrai (urban: 14.9% in 2001 to 5.0% in 2009; rural: 5.2% in 2001 to 1.3% in 2009) [14].

As in most of Ethiopia, in Tigrai male circumcision is almost universal at 96.2% in the region [7] and is likely to have affected the transmission dynamics and contributed to maintaining a relatively low level of the epidemic as shown in other contexts in Sub-Saharan Africa [15-17].

### A clear rural–urban divide in HIV prevalence

While all urban areas invariably experienced the epidemic, rural HIV prevalence was consistently low. According to 2009 ANC surveillance data HIV prevalence in Tigrai was 5.0% in urban areas and 1.3% in rural (2.2% overall) [14]; while according to EDHS 2011 it was 4.2% among urban females versus 1.5% among rural females and 1.5% among urban males versus 1.2% in rural males. Similarly, analysis of routine PMTCT testing data from 2008/09 to 2010/11 shows consistently lower HIV prevalence in rural areas (0.9% in 2008/09, 1.3% in 2009/10 and 0.3% in 2010/11). However, potential epidemics (based on routine PMTCT testing data; prevalence >3.5%, up to 7.3% in Alamata Town and up to 5.1% in rural Raya Azebo in 2010) were seen in rapidly urbanizing economic zones and commercial farm districts of western and southern Tigrai. This observation has also been reported in a previous study amongst rural agricultural communities in Ethiopia including Tigrai [18].

### HIV and AIDS heterogeneity by administrative zones

**Figure 2:**
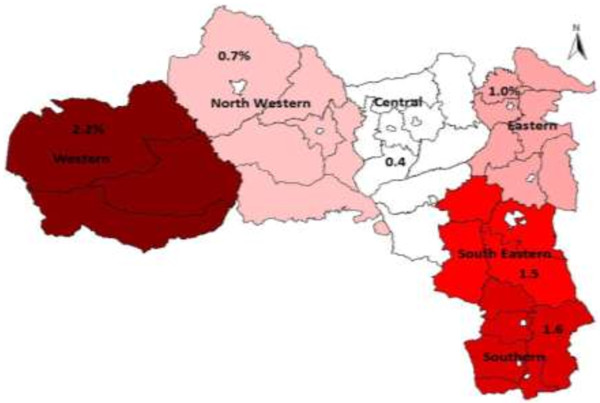
The HIV epidemic in Tigrai by administrative zone, Tigrai 2010/11, PMTCT HIV testing data (ANC routine) (THB profile).

According to PMTCT routine testing data for 2010/11 the prevalence of HIV in Tigrai was found to be higher in the western zone (2.2%), due to the presence of commercial farming with high numbers of male seasonal workers and related higher risk behaviours such as unprotected transactional sex (as reported in FGDs). This is followed by southern and south eastern zones (1.6% and 1.5% respectively) most likely due to the presence of large towns and since these are growing economic zones that host a major transport route to the capital that are also conducive for risky sexual behaviours such as commercial sex and multiple partnerships. In contrast, lower prevalence of HIV was found in the central zone (0.4%) [19] (Figure 2).

### HIV/AIDS heterogeneity by administrative district

**Figure 3:**
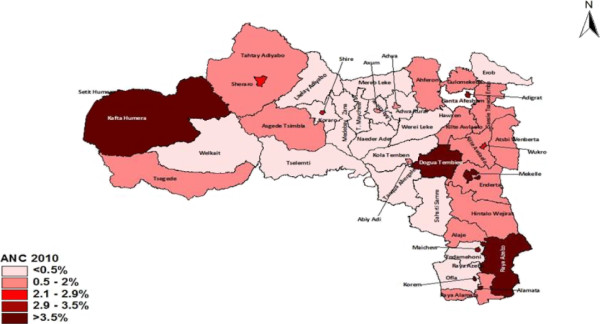
The HIV epidemic by administrative district, Tigrai 2010/11, PMTCT HIV testing data (ANC routine).

Differences in HIV prevalence based on 2010/11 PMTCT routine testing data are clearer when prevalence at a lower administrative level (the district) is considered (Figure 3). A higher prevalence can be seen in urban districts, particularly in the regional capital Mekelle (4.2%) as well as other urban centres such as Alamata town (7.3%), Korem (4.6%) and Setit Humera town (3.7%). Higher prevalence is also found in districts along the main road from Alamata to Adigrat and Adwa to Enda Selase Shire. This is the most accessible part of Tigrai with a number of urban areas including Mekelle and a high concentration of economic activity. Moreover high HIV prevalence was observed in Kafta Humera (4.3%), a district with a large population of seasonal workers, and in the district of Raya Azebo (5.1%), a designated economic zone where there are large scale commercial farms. Higher prevalence was also observed in the part rural and part peri-urban district of Degua Tembien (3.8%), a feature which could not be fully explained.

### HIV risk factors

**Figure 4:**
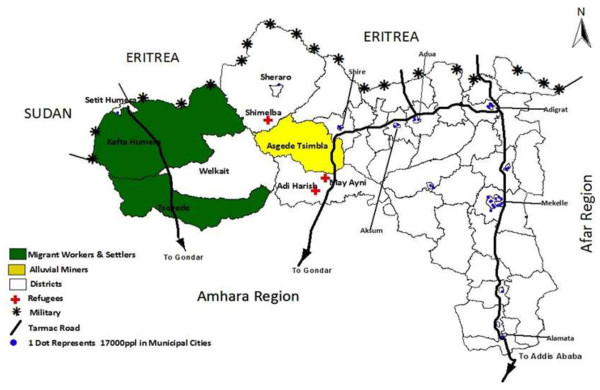
Map of HIV risk factors in Tigrai.

Some of the risk factors that may contribute to the variation in HIV prevalence in Tigrai are shown in Figure 4 and include the following: the main tarmac roads passing through urban settlements; areas where there is a concentration of seasonal workers and commercial farms and areas with a presence of alluvial miners. An overall correspondence between Figure 3 (HIV prevalence at district level) and Figure 4 (the presence of risk factors for HIV) is noticeable. Other potential risk factors, such as the presence of refugee camps and the deployment of army troops were not confirmed to have had a particular influence on the epidemic in the region.

As summarised below, for each of the key population groups we looked at HIV prevalence, risk behaviours/vulnerability and estimated size of the population group. We then classified the evidence according to its strength. All of these factors are essential while assessing the potential role of a population group in the epidemic dynamics and while prioritizing HIV prevention services.

### Young women (15–24 years)

Routine HCT and PICT data for Tigrai from 2010/11 show HIV prevalence in young women to be twice that of young men (HCT: 1.0% in females versus 0.4% in males; PICT: 1.4% versus 0.7%). This has been ascribed to the tendency (EDHS data) for girls to have sexual debut at an earlier age than boys, or have older partners. However, the 2011 EDHS estimated the prevalence of HIV among women and men aged 15–24 years in Tigrai to be almost equal: 0.6% and 0.7% respectively. The sample size for this finding however was small. As with the rest of Ethiopia, in Tigrai sexually active, never-married women were at much higher risk of being HIV positive (2.8%) than never-married men (1.6%) [7]. There is no data in Tigrai on prevalence in specific youth population groups that may be at higher risk of HIV, namely university students, orphans and vulnerable children (OVCs) and street children.

### Seasonal workers

Health facility data from several HCT testing sites in areas where seasonal mobile workers are present found HIV prevalence ranging from 0.5% to 8.2%. Moreover, the size of this group is large with an estimated 200,000 seasonal workers [20], approximately 15% of the adult population of Tigrai [21], and may therefore have a strong influence in driving the epidemic in the region. FGDs conducted for this study with seasonal workers revealed that seasonal migrant workers tend to be young and highly mobile men often separated from their families for long periods of time. Reportedly the young males frequent sex workers more often than married males, and many do not use condoms despite being aware of the risks of HIV.

### Sex workers

Available data vary in quality but all point to a high prevalence, ranging from 10% to 20% [HIV prevalence among HCT attendees, key populations. Unpublished document, Routine HIV Counselling and Testing data. Unpublished records] which is several times higher than the overall population prevalence. There has been no size estimation of sex workers in Tigrai and hence the size of the sex worker population remains unknown. In terms of behavioural data limited evidence from a recent survey of mobile VCT clients indicates that sex workers are increasingly using condoms regularly with paying clients, but not with non-paying partners [22]. FGDs conducted as part of this study found the clients of sex workers varied and included soldiers, daily labourers, truck drivers and migrant workers. Sex workers reported some clients resisted condom use and used violence to avoid using condoms.

### Discordant couples

The number of discordant couples was estimated at about 10,000 (based on population data from the 2007 population census [21] and EDHS 2011 data on discordancy [7]). FGDs with people living with HIV indicated low condom use and low levels of disclosure pointing to high exposure risks for HIV negative partners.

### Other key populations

Despite the assumption of vulnerability and reported behaviours during FGDs that would place them at higher risk, HIV prevalence seems to be low in other key populations, not being appreciably different from the general population prevalence although sample sizes for available studies were small. This was true for truck drivers, refugees and prisoners. According to a recent study on HCT attendees, HIV prevalence among truck drivers in Tigrai was 0.4%, however, only 228 long distance truck drivers were sampled [HIV prevalence among HCT attendees, key populations. Unpublished documents]. Similarly HIV prevalence among refugees in two camps in Tigrai (Shimelba and Mai Ayni) was also low: 0.5% according to HCT data in 2010/11 and 0.4% according to 2010/11 PMTCT HIV routine testing data for both camps. Looking at HCT and VCT data from Mekelle prison, it seems that HIV prevalence among inmates and prison staff does not differ much from the general Tigrai population (VCT 2011 data: 1.3%; HCT 2011 data: 2.4%) [Routine HIV counselling and testing data, July to September 2011. Unpublished records, Routine HIV Counselling and Testing data. 2011. Unpublished]. Additional potential high risk groups for which no data exists includes men who have sex with men (MSM), injecting drug users (IDUs) and OVCs/street children. In the case of alluvial miners, university students and uniformed services (the military and police forces) it was not possible to conclude on their level of HIV risk due to paucity of data and/or conflicting results from available studies [HIV prevalence among HCT attendees, key populations. Unpublished document, [23,24]. FGDs conducted with these groups as part of this study, however identified various risk behaviours including inconsistent condom use, multiple partners and interactions with sex workers.

### The HIV response in Tigrai

The most recent strategic plan of the Tigrai HIV prevention and control office [25] covers basic HIV services in prevention, treatment, care and support in line with the national strategic plan for the multisectoral HIV response [26].

### Health system strengthening

In 2005 the number of health centres in the region was only 42 and most of these were located in urban and peri-urban areas. By 2010, the number rose to 202, as a result of the construction of an additional 160 health facilities mostly in rural districts [19].

### Behaviour change communication (BCC)

Efforts to raise awareness and conduct behavioural change communication were carried out mainly by training a team of health staff for every health centre and via mass media including local radio stations and newspapers. The health extension programme, the Ethiopian version of primary health care, has also been utilised for BCC for HIV at the household level. Health extension workers are deployed at the health post level primarily in rural areas. Community conversation is yet another key programme where members of a community come together to discuss health and HIV related issues and develop a plan of action to address them. By 2011, the Federal HIV Prevention and Control Office reported that all 714 districts of Tigrai had a community conversation programme in place [27].

KIIs uniformly reported that these efforts are likely to have contributed to the higher levels of comprehensive knowledge about HIV and AIDS (correctly identify two ways to prevent HIV and reject 3 HIV related misconceptions) in Tigrai which is above the national average (females: Tigrai 22.1% vs. 18.5% Ethiopia, p < 0.0001; males: Tigrai 41.0% vs. 31.1%, Ethiopia, p < 0.0001), with improvement seen in each individual knowledge indicator between 2005 and 2011 [28,7].

### HIV counselling and testing services

The number of HIV testing sites in Tigrai increased significantly between 2003 and 2011 (from 23 sites to 274). Similarly there was a dramatic rise in the uptake of testing, from less than 6,000 people tested in 2003/04 to over one million in 2010/11 alone [29,27]. This is also supported by EDHS 2011 which reported that 55.5% of women and 49.1% of men 15–49 years of age in Tigrai were tested for HIV. This is one of the highest rates in the country [7]. HCT is an entry point to other services such as ART and could have potentially contributed to the observed increase in ART uptake as reported below.

### Prevention of mother to child transmission

PMTCT programme implementation began in Tigrai in 2002. In 2005 the Tigrai Health Bureau had nine PMTCT sites which grew to 186 sites in 2011. HIV testing uptake by mothers who attended ANC showed a steady increase rising from 4,493 in 2005/06 to over 100,000 in 2010/2011. However, the PMTCT coverage of HIV positive pregnant women remains as low as 24%, and progressive loss of pregnant women is seen at every step of the PMTCT cascade, beginning with non-attendance at ANCs [27,30] (ANC coverage^a^ was 50.1% in Tigrai in 2012 [7]). In 2010/11, 37% of pregnant women attending ANCs were not tested for HIV and 52% of identified HIV positive pregnant women did not receive ARV prophylaxis. In fact, many HIV positive pregnant women do not deliver at health facilities [27].

### Antiretroviral treatment

Since the introduction of free provision of ART in 2005, the number of sites offering ART and people on treatment has risen rapidly (from 4 in 2005 to 77 in 2011) [30,27]. About 30,000 people were estimated to be in need of ART (at CD4 < 200) in Tigrai in 2011, and corresponding coverage was estimated at 70% [13]. However, of the total number of patients who had ever started ART, only 73% (20,874) were currently on ART in 2010/11 [27].

### Combined HIV prevention for key population

Some limited HIV prevention services are available for sex workers, but these mainly focus on income generating activities (IGAs) - for which evidence on effectiveness is inconsistent [Review of the evidence base for an “evidence based” policy on HIV programming with sex workers*.* 2007. Unpublished document]; the same is true for OVCs. Similarly, although there are a few limited HIV services, a comprehensive and consistent package of HIV prevention for truck drivers is lacking. Services for seasonal workers focus on ART only. For prisoners, the minimum HIV services covering BCC, HCT, treatment and care are available but no condom provision. For in school youth and university students some HIV programmes are in place including BCC but these need strengthening. No specific HIV services exist for IDUs, MSM, alluvial miners, and street children. Despite the evidence, there are also no services for discordant couples. In contrast, the HIV prevention response seems adequate for military, police and refugees as these groups receive special attention from government and partners. Some HIV mainstreaming workplace programmes in the public sector are in place for employees, families and surrounding communities but no data was available on HIV work place interventions in the private sector.

### Resource allocation

Data was not available to enable a detailed analysis of resource allocation. The only HIV spending assessment conducted in Ethiopia at the time of this study was a National Health Accounts survey for 2007/8 [31]. However, findings were not disaggregated by region. Review of funds received by Tigrai HAPCO between 2004 and 2010 revealed that three quarters of funds were used for HIV prevention (13 million USD). However, funds allocated for construction of primary health care units which offer HCT, PMTCT and ART services, inflated expenditure on prevention. Moreover the funds did not capture support received from certain major HIV funders. There is little evidence to suggest resources are prioritized towards targeting population groups who are more vulnerable to HIV infection. However it is worth noting the activities from civil society organisations that implement HIV programmes for key populations are not captured in the resource tracking system of Tigrai HAPCO.

### Study limitations

The major limitation of this study was the paucity of data and the limited quality. There is a lack of information on key populations, for which there are neither size estimates nor representative studies on HIV prevalence. There is also limited knowledge, attitudes, practice and behavioural data available. This necessitated use of data from smaller studies which have methodological limitations and are difficult to generalise from. Moreover this study utilised routine data collected over a number of years (2008/2009 - 2010/2011) [2004, 2005, 2006, 2007, 2008, 2009, 2010, 2011: Annual profiles*.* Unpublished documents]. But routine data is often incomplete and may be biased. The major advantages of using routinely collected, facility-based data for HIV epidemic analysis include: lower cost, collection of some individual level demographic data in addition to HIV prevalence; and high coverage of the population. Efforts were made to address these limitations by triangulating and synthesising data from multiple sources including EDHS, ANC sentinel surveillance, routine HIV health facility testing data and qualitative data from FGDs with key populations and KIIs with HIV programme implementers and policy makers. This served to make sense of the diverse information on HIV in the region by looking for convergence of data and to highlight priority information gaps for future study where indication for risks exist but actual evidence was weak.

The incidence model projecting new infections (Modes of Transmission model) could not be used because the population estimates of several populations groups at higher risk of HIV could not be assessed and because of a lack of verifiable HIV prevalence and behavioural data for each key population group.

## Conclusions

Our results demonstrate the importance of utilizing routine data on the HIV epidemic and response at sub-national level in order to obtain meaningful information for a targeted HIV response. This is true for countries as large and heterogeneous as Ethiopia.

By taking a granular approach, we were able to identify elements in the HIV epidemic that were previously unknown as well as validate and substantiate existing unverified observations and trends. Local stakeholders were able to provide strong interpretation for variations in HIV prevalence for a deeper and more operational understanding of the HIV epidemic in and within the region.

In view of the main findings of this study the following recommendations were suggested to address the issues or challenges identified following assessment of the degree of alignment between the HIV response and the nature of the epidemic in Tigrai.

### Strategic information

There is a lack of comprehensive strategic information on key populations. Data on HIV prevalence and size estimates for high risk population groups are not available to inform and guide HIV prevention. It is recommended to introduce representative prevalence and behavioural studies on key populations, starting with sex workers, mobile seasonal workers and miners, given their large numbers, risk profiles and higher HIV prevalence than the general population.

Moreover, a lack of knowledge at the regional level on how overall funds are allocated to different services and locations, the source of the funding, or the accounting of expenditures was observed. Therefore, it is recommended that future surveys of expenditures carried out at the national level should be disaggregated by regions. In addition, development partners should be accountable for reporting on funding at the regional level so that this can be accounted for during regional programme planning and Tigrai HAPCO should take a stronger role in coordinating and monitoring HIV resources.

### Programming for key populations

There are few programmes in operation to address the needs of high risk groups including sex workers, alluvial miners, seasonal farm workers, discordant couples and long distance drivers. Thus to address this the following is recommended: i) while further research is needed to estimate the size and HIV prevalence of these high-risk groups, programmes should be started while research is ongoing on the assumption that there is a demonstrable need; ii) implement the minimum package of services for high-risk populations [32] and iii) establish systematic surveillance of HIV prevalence for all high risk groups. Existing programmes that address issues in key populations need to be strengthened and expanded. Joint programming efforts by government and civil society organisations are needed to reach these populations. In particular, there is a need to prioritise provision of comprehensive and tailored service packages for sex workers and their client populations along the transport corridor of east and south eastern Tigrai, and to provide comprehensive workplace interventions in companies with seasonal, migrant and mobile labour (mainly western Tigrai). Discordant couples are a particular high risk group which should be given greater attention. It is recommended that targeted services for discordant couples within the health system, including those focusing on positive prevention are developed. Such programmes should go beyond clinical services and cover impact mitigation, including positive living with HIV, safe sex and family planning.

### Programming according to geographical needs

Despite the variation in HIV prevalence there is no evidence that programme planning is done on the basis of geographical considerations and thus HIV needs. Therefore, it is recommended that i) programme planning at zonal or district level should factor in differences in HIV prevalence in addition to population size ii) a study in Degua Tembien should be conducted to determine factors accounting for the unexplained higher HIV prevalence and iii) while HIV programmes should be focused on urban areas, programming should not neglect those rural areas that are of higher prevalence, or where location such as proximity to transport corridors or high-risk populations place the local population at risk.

### PMTCT

Follow up of the PMTCT programme is inadequate so much so that of the pregnant mothers that test positive, less than half receive ARV prophylaxis/ARV treatment; in addition, less than half of their babies received ARV prophylaxis. Within the global commitment to eliminate mother-to-child transmission, the PMTCT programme needs to be strengthened, to ensure that all pregnant women who attend ANC are tested and followed up to delivery and through the breastfeeding period, and that they and their newborns receive appropriate services.

### Antiretroviral treatment

ARV-based interventions (ART for individuals with CD4 count <500 and PMTCT) can make a key contribution to further reduce HIV transmission [33], and respective service coverage, adherence and quality issues should be a focus of the Tigrai health sector. Moreover ART at quality and scale reduces morbidity, mortality [34] and orphanhood and thereby reduces future care and support costs [35].

We believe the above recommendations are in line with the new Investment Framework that supports a more prioritised, efficient and effective HIV response based on country context to maximise benefit [36]; and, if implemented, will strengthen efforts towards reaching zero new HIV infections. The key lesson learned from this HIV synthesis exercise was the current lack of focused response. In Tigrai region the available evidence suggests the epidemic is declining and becoming more concentrated in certain areas and key populations. Thus going forward HIV investments should target these groups. For instance, in spite of the high risk amongst seasonal farm workers in the commercial farms, there was at the time of the study, no HIV interventions or services targeting these groups. Discordant couples, a group that has been overlooked by the HIV response, were finally recognized as a priority for HIV prevention due to the exposure risks HIV negative partner’s face. This study contributes to the local HIV response which maximises impact and breaks from the “business as usual” model of spreading HIV resources too thinly over too many objectives.

For a more effective and targeted HIV prevention response, studies and data syntheses need to be carried out at a granular/sub-national level in order to accurately identify local specificities and plan accordingly. This study is the first HIV synthesis carried out at regional level in Ethiopia. Following the example of Tigrai region, other regions of the country are now carrying out similar exercises that complement, deepen and clarify findings from national syntheses to better tailor their response. Other countries with large populations that are heterogeneous in relation to HIV should also consider sub-national approaches.

## Endnote

^a^Defined as the proportion of women aged 15–49 years who had a live birth in the 5 years preceding the 2011 demographic and health survey, receiving antenatal care from a skilled provider.

## Abbreviations

AIDS: Acquired immunodeficiency syndrome; ANC: Antenatal care; ARV: Antiretroviral; ART: Antiretroviral therapy; BCC: Behaviour change communication; CI: Confidence interval; EDHS: Ethiopian demographic and health survey; FGD: Focus group discussion; HAPCO: HIV prevention and control office; HCT: HIV counselling and testing; HIV: Human acquired immunodeficiency syndrome; IDU: Injecting drug user; IGA: Income generation activities; KI: Key informant; KYE: Know your epidemic; KYR: Know your response; MSM: Men who have sex with men; NGO: Non-governmental organisation; OR: Odds ratio; OVC: Orphans and vulnerable children; PICT: Provider initiated testing; PMTCT: Prevention of mother to child transmission; UNAIDS: Joint United Nations Programme on HIV/AIDS; VCT: Voluntary counselling and testing.

## Competing interests

All authors declare they have no competing interests. The findings and conclusions of this paper are those of the authors and do not necessarily represent the views of the funders of this work.

## Authors' contributions

GB led the study, undertook the data collection and drafted the article. EP and HH conducted the analysis of the quantitative and qualitative data and contributed to drafting the paper. SZ and WN facilitated the conduct of the study and provided overall guidance for the conception and implementation. All authors read and approved the final manuscript.

## Pre-publication history

The pre-publication history for this paper can be accessed here:

http://www.biomedcentral.com/1471-2458/14/628/prepub
